# Research priorities to strengthen environmental cleaning in healthcare facilities: the CLEAN Group Consensus

**DOI:** 10.1186/s13756-024-01463-9

**Published:** 2024-09-27

**Authors:** Giorgia Gon, Angela Dramowski, Emilio Hornsey, Wendy Graham, Nasser Fardousi, Alexander Aiken, Benedetta Allegranzi, Darcy Anderson, James Bartram, Sanjay Bhattacharya, John Brogan, An Caluwaerts, Maria Clara Padoveze, Nizam Damani, Stephanie Dancer, Miranda Deeves, Lindsay Denny, Nicholas Feasey, Lisa Hall, Joost Hopman, Laxman Kharal Chettry, Martin Kiernan, Claire Kilpatrick, Shaheen Mehtar, Christine Moe, Stephen Nurse-Findlay, Folasade Ogunsola, Tochi Okwor, Bruno Pascual, Molly Patrick, Oliver Pearse, Alexandra Peters, Didier Pittet, Julie Storr, Sara Tomczyk, Thomas G. Weiser, Habib Yakubu

**Affiliations:** 1 London School of Hygiene and Tropical Medicine, London, UK; 2 Stellenbosch University, Stellenbosch, South Africa; 3 UK Health Security Agency, London, UK; 4 Infection Prevention and Control Unit, Integrated Health Services, World Health Organisation (WHO) HQ, Geneva, Switzerland; 5 The Water Institute, University of North Carolina at Chapel Hill, Chapel Hill, USA; 6 University of Leeds, Leeds, UK; 7 Tata Medical Center, Kolkata, India; 8 Helvetas, Zurich, Switzerland; 9 IPC Consultant, Brussels, Belgium; 10 Universidade de São Paulo, São Paulo, Brazil; 11 Southern Health and Social Care Trust, Portadown, UK; 12 NHS Lanarkshire, Bothwell, UK; 13 World Health Organization, Geneva, Switzerland; 14 United Nations Children’s Fund, New York, USA; 15 Liverpool School of Tropical Medicine, Liverpool, UK; 16 Malawi-Liverpool-Wellcome Trust Clinical Research Programme, Blantyre, Malawi; 17 University of Queensland, Brisbane, Australia; 18 Radboud University Nijmegen Medical Centre, Nijmegen, Netherlands; 19 Terre des Hommes, Geneva, Switzerland; 20 Richard Wells Research Centre, University of West London, London, UK; 21 KSHealthcare Consulting, Glasgow, UK; 22 Infection Control Africa Network, Cape Town, South Africa; 23 Emory University, Atlanta, USA; 24 University of Lagos, Lagos, Nigeria; 25 Nigeria Centre for Disease Control, Abuja, Nigeria; 26 Centers for Disease Control and Prevention, Atlanta, USA; 27 University of Geneva, Geneva, Switzerland; 28 Robert Koch Institute, Berlin, Germany; 29 Stanford University, Stanford, USA; 30 Edinburgh Napier University, Edinburgh, UK

**Keywords:** Environment cleaning, Healthcare facilities, Research priorities

## Abstract

Environmental cleaning is essential to patient and health worker safety, yet it is a substantially neglected area in terms of knowledge, practice, and capacity-building, especially in resource-limited settings. Public health advocacy, research and investment are urgently needed to develop and implement cost-effective interventions to improve environmental cleanliness and, thus, overall healthcare quality and safety. We outline here the CLEAN Group Consensus exercise yielding twelve urgent research questions, grouped into four thematic areas: standards, system strengthening, behaviour change, and innovation.

## Background

It is now well over a century since hygiene pioneers, like Florence Nightingale, introduced the importance of cleanliness to the healthcare community. This is highlighted in the 2023 joint global report by the World Health Organization (WHO) and UNICEF on the state of hygiene services in healthcare facilities [1]. The report flags huge gaps in knowledge, practice, and capacity building for environmental cleaning, here defined as the application of water and detergent, and disinfectant where necessary, to surfaces and non-critical equipment by cleaning staff [1, 2]. These gaps have serious consequences for both patient and health worker safety, worldwide and in particularly in resource-limited settings [1, 3]. For example, of the hospitals participating in the 2023 joint report from the Eastern Mediterranean region only a third reported the presence of any formal training programmes for cleaning staff and the availability of cleaning protocols. Moreover, access to cleaning materials in low-income countries was only half of that found in high-income countries [1]. The 2023 report also highlights the paucity of data on environmental cleanliness and, hence, the inability to produce global or regional estimates. Other studies have reported high levels of surface contamination in patient areas in hospitals in low and middle-income countries [4–6]. Surface contamination is plausibly linked with healthcare-associated infections (HAIs) [7–9] because microorganisms on surfaces, such as surfaces in the bed area, are transmitted to patients directly, and also indirectly by hands or via equipment used by health workers, patients or visitors [10]. Emerging evidence about airborne dissemination leading to contamination of surfaces and people is a further consideration. The relative contribution of the environment to the burden of HAIs is uncertain—possibly contributing to 20% of the transmission [11]—and is likely to be context-dependent. Rigorous studies into achieving environmental hygiene using cost-effective interventions are few [7, 12, 13]. Bridging this gap offers opportunities to improve environmental cleanliness and, thus, overall healthcare quality and safety.

## Main text

This commentary summarises the recommendations of the CLEAN Group Consensus exercise in the field of environmental cleaning; the full briefing is available at: https://media.tghn.org/medialibrary/2023/06/11730_LSHTM__CleanBriefing_PDF_FINAL_07042023.doc.pdf. [12] Convened by UK-PHRST, a multi-disciplinary stakeholder group (the CLEAN Group) was engaged to identify the most urgent and current implementation research questions focusing primarily on resource-limited settings. The group had participants from Africa, Europe, Western Pacific, Asia, North and South America with research expertise in infection prevention and control, cleaning and disinfection, health policy, and implementation science.

Between March and October 2022, the CLEAN Group followed a systematic prioritisation process using the REPRISE guidelines [14] The 12 priority research questions identified fell into four thematic areas: standards, system strengthening, behaviour change, and innovation. For example, one question is *“What are the health system-level factors that can support the professionalisation of cleaning staff?”* By “professionalisation of cleaning staff”, we refer to the process of ensuring that cleaning procedures are performed by trained staff who are skilled and work with fair contractual arrangements that allow them to perform their duties with dignity and to participate in decision-making. In most contexts, cleaning staff are predominantly women and of low socioeconomic status [15, 16], and in some settings, ethnicity and other characteristics of self-identity also affect their status and treatment. These identities, cleaners’ self-agency and their limited autonomy, all intersect and impact on improving environmental cleaning. Research is urgently needed to explore options for the professionalisation of cleaning staff and with the full engagement of cleaning staff themselves. Empowering cleaning staff is part of the health services’ duty of care to keep patients, visitors and health workers safe.

Answering the 12 research questions highlighted by the CLEAN Group [17] would facilitate progress on universal quality of care, and universal coverage of safely managed drinking water, safely managed sanitation, and basic hygiene services (respectively Sustainable Development Goal 3 and 6). Environmental cleaning programmes should be seen as a critical part of health systems and should be aligned with the global and local calls to action to ensure quality and safety, such as achieving the WHO Infection Prevention and Control Core Components and global action plan and the Antimicrobial Resistant Global Action plan [18–20]. Indeed, successful cleaning programmes can only be achieved if their management, transparency and accountability are a priority at the institutional and health system levels [18, 21].

Beyond the priority questions, the CLEAN Briefing [12] also makes broader recommendations for implementation research in environmental cleaning. For example, there is a pressing need to have cleaning benchmarks. There are currently no internationally recognised standards for thresholds of cleanliness which demarcate unacceptable levels of risk of HAIs, and current suggested cleaning routines are based on weak evidence. A further example is the need for research to contextualise environmental cleaning guidance, allowing for such factors as who has cleaning responsibilities and under what working conditions, whether services are contracted out, levels of human resources (numbers by levels of training and roles), access to clean water, sanitation and hygiene infrastructure, conditions and materials of items to be cleaned, cleaning supplies, patient flow and the wider facility-level organisational aspects to ensure accountability of environmental cleaning programmes.

## Conclusions

In 1885, the London Times exposed the consequences of unhygienic conditions in military hospitals. Despite the intervening 139 years, cleanliness in healthcare facilities is still widely deficient, with adverse consequences for healthcare systems, budgets, and foremost—for the safety of patients and staff. Funders, policymakers and researchers can all play key roles in advancing the research agenda presented in the CLEAN Briefing, and ultimately ensuring cost-effective and contextually appropriate interventions are implemented to accelerate the much needed progress in this field.

## Data Availability

The article discussed is available at https://media.tghn.org/medialibrary/2023/06/11730_LSHTM__CleanBriefing_PDF_FINAL_07042023.doc.pdf).
